# Resolving different presynaptic activity patterns within single olfactory glomeruli of *Xenopus laevis* larvae

**DOI:** 10.1038/s41598-021-93677-9

**Published:** 2021-07-09

**Authors:** Rodi Topci, Mihai Alevra, Erik H. U. Rauf, Daniëlle de Jong-Bolm

**Affiliations:** 1 Institute for Neurophysiology and Cellular Biophysics, University Medical Center Göttingen, 37073 Göttingen, Germany; 2 Department of Neuro- and Sensory Physiology, University Medical Center Göttingen, 37073 Göttingen, Germany; 3 Center Nanoscale Microscopy and Molecular Physiology of the Brain (CNMPB), Göttingen, Germany

**Keywords:** Olfactory system, Olfactory bulb, Neurophysiology

## Abstract

Olfactory sensing is generally organized into groups of similarly sensing olfactory receptor neurons converging into their corresponding glomerulus, which is thought to behave as a uniform functional unit. It is however unclear to which degree axons within a glomerulus show identical activity, how many converge into a glomerulus, and to answer these questions, how it is possible to visually separate them in live imaging. Here we investigate activity of olfactory receptor neurons and their axon terminals throughout olfactory glomeruli using electrophysiological recordings and rapid 4D calcium imaging. While single olfactory receptor neurons responsive to the same odor stimulus show a diversity of responses in terms of sensitivity and spontaneous firing rate on the level of the somata, their pre-synaptic calcium activity in the glomerulus is homogeneous. In addition, we could not observe the correlated spontaneous calcium activity that is found on the post-synaptic side throughout mitral cell dendrites and has been used in activity correlation imaging. However, it is possible to induce spatio-temporal presynaptic response inhomogeneities by applying trains of olfactory stimuli with varying amino acid concentrations. Automated region-of-interest detection and correlation analysis then visually distinguishes at least two axon subgroups per glomerulus that differ in odor sensitivity.

## Introduction

The anatomy of olfactory receptor neurons (ORNs) from *Xenopus laevis* is comparable to those of mammals. Their somata are embedded in the olfactory epithelium, their axons form the olfactory nerve and their terminals converge into the olfactory glomeruli that lay in the olfactory bulb^1^. A common assumption among species is that axons from multiple ORNs (presynaptic side) as well as dendrites from multiple mitral cells (post-synaptic side) converge into a single glomerulus^2–4^. Even so, until today, except for some ratios, there are no estimations about the number of ORNs or mitral cells that terminate in a glomerulus. For example, the ratio between ORNs and mitral cells in *Xenopus laevis* is estimated to increase over development, ranging from 5:1 in the developmental stages 50–58 up to 34:1 in adult frogs^5^. For mice, the number of glomeruli is estimated at 1.810 ± 41, with a corresponding number of 1000–2000 ORNs per glomerulus^6^.

The numerous ORNs that converge into a single glomerulus express the same odorant receptors^6–10^. Consequently, calcium imaging within single glomeruli revealed similar calcium patterns, despite small variations^11^. Larger variations were found during development^12^ or in mice lacking olfactory marker protein (OMP^−/−^), but not in wild type mice^13^.

Variable ORN response shapes after repetitive odor exposure are common, especially when using high concentrations or short inter-stimulus intervals^14^. Also developmental stage^15^ and endocannabinoid levels^16^ play a role in shaping odor responses. Since response variations appear regularly on the single cell level, it is not surprising that in line with this large dynamic ranges are observed among ORN populations across species^17,18^ even when they have the same odorant receptor^19,20^.

Therefore, we decided to investigate the similarity and variability of activity patterns of ORNs. We hypothesized that rapid 4D calcium imaging in the olfactory bulb (OB) would reveal distinguishable intraglomerular variations as a representation of ORN variability in the olfactory epithelium.

Dose–response experiments under rapid imaging conditions revealed different sensitivities to the same odors among individual regions from a single glomerulus. These observations were in line with complementary patch-clamp recordings at the level of the olfactory epithelium.

Based on both electrophysiological and calcium imaging recordings, we developed an odor-stimulation protocol to further separate intraglomerular axon terminals originating from different ORNs. For those optimized conditions, a semi-automatized ROI selection in combination with a correlation-based analysis enables the detection and visualization of intraglomerular axonal projections with different activity patterns (typically two).

Although our results confirm that calcium activities from intraglomerular axon terminals are similar, they are different and distinguishable with respect to sensitivity, which implies that intraglomerular axonal branches are functionally distinct.

## Results

### Initial recordings: selection of anatomical and functional glomeruli

**Figure 1:**
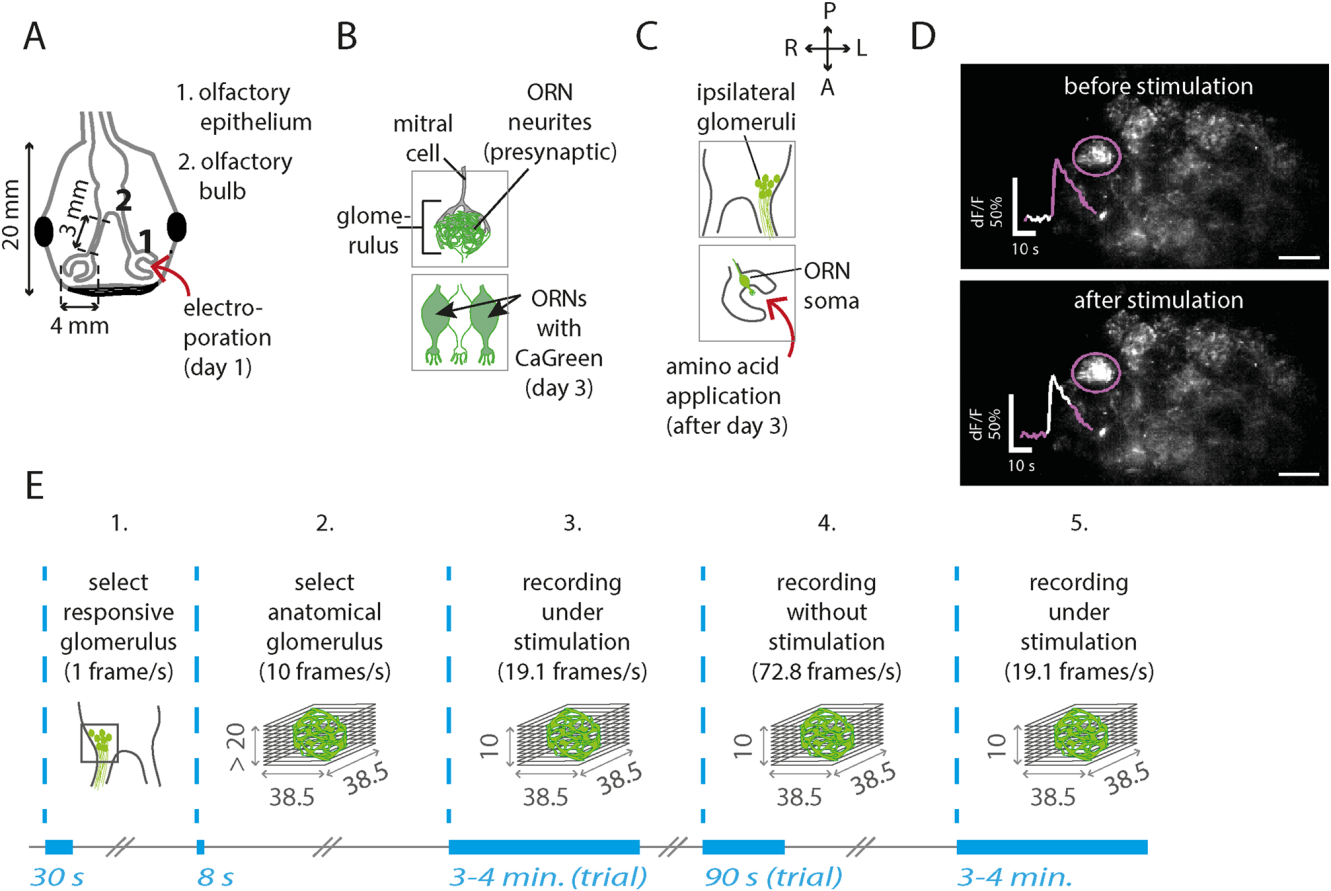
Localizing amino-acid responsive glomeruli in the lateral olfactory bulb of *Xenopus laevis* tadpoles. (**A**) Drawing of typical tadpole head. It shows the location of the olfactory epithelium (1) and the olfactory bulb (2). In this study, the olfactory epithelium was electroporated to bring CaGreen into ORNs and their axon terminals. (**B**) After electroporation, many ORNs contain the calcium sensor CaGreen. Since ORN neurites form the presynaptic side of glomeruli, it is possible to record calcium responses in the olfactory bulb 3 days after electroporation. Mitral cell dendrites form the post-synaptic side of glomeruli. Both neuron types have branches that project to the same and to other glomeruli. (**C**) Glomeruli are found in clusters. The lateral cluster consists of amino-acid responsive glomeruli. The rapid 4D recordings shown in this study were obtained from the ipsilateral cluster. *A* anterior, *P* posterior, *M* medial, *L* lateral. (**D**) Illustrative example of an amino-acid responsive glomerulus from the ipsilateral cluster. For stimulation, an amino-acid mixture was used (concentration 10 µM). Scale bar: 10 µm. (**E**) Experimental design of rapid 4D calcium imaging. Start and duration of each recording is shown in blue. **1:** Single plane recording to select a glomerulus that responds to a 10 µM or 100 µM amino-acid mixture. **2:** z-stack of a responsive glomerulus to get an impression of its anatomical borders and its core. Inter-plane distance: 1 µm. **3 and 4:** Recording under stimulation or without stimulation. Repetitions of those steps are indicated as trials. The center of the glomerulus lies approximately between the fifth and sixth z-plane. **5:** The last stimulation should reveal a response and the corresponding z-stack should contain only minimal morphological changes. If so, trials were considered as suitable for analysis. Matlab 2017b and Adobe Illustrator CS6 were used for composition of this figure (see “Methods” for further details about the used software).

To obtain calcium-imaging recordings from single glomeruli of *Xenopus laevis* tadpoles, the calcium sensor Calcium Green-1 dextran and the experimental design shown in Fig. 1 was used. Calcium Green-1 dextran was brought into the ORNs at least 3 days before (recovery time) via local electroporation at the level of the olfactory epithelium.

Glomeruli from the lateral glomerular cluster have the glomerulus-specific spherical structure and respond to amino acids, which are their adequate stimuli^21^. Therefore, to validate the shape and responsiveness of a glomerulus, two initial calcium-imaging recordings were performed. During the first measurement (ipsilateral to the odor application side), we follow the calcium sensor responsiveness from ORN axon terminals in single glomeruli under the application of a mixture of amino acids [10 µM or 100 µM; Fig. 1C–E (step 1)]. Amino-acid-responsive ORN axons were considered as being functional in our assay. The second measurement was a detailed z-stack of an area with functional ORN axons (Fig. 1E, step 2). This z-stack was necessary in order to confirm that responsive axons were indeed part of a spherical neuropil-like structure, reflecting the typical spherical anatomy of olfactory glomeruli. Such a z-stack covered a depth of at least 20 µm with a distance of 1 µm between planes (Fig. 1E, step 2). When both criteria were met, the glomerulus was considered as a functional and anatomical neuropil and selected for rapid 4D calcium-imaging recordings.

### Rapid 4D calcium imaging recordings: region-specific calcium fluctuations within single glomeruli

We expected that calcium imaging in the olfactory bulb (OB) on the one hand would confirm typical features of glomeruli, such as having similar odor responses and responding to odor concentrations over multiple log scales. On the other hand, we expected the previously observed multidimensionality on the level of the olfactory epithelium to be passed on to the intraglomerular axon terminals in the OB. Accordingly; we hypothesized that rapid 4D calcium imaging would reveal distinguishable fluctuations within single glomeruli as well. To test this hypothesis, glomerular response profiles and spontaneous activity were obtained with our custom-built line-illuminating microscope using time-series of z-stacks (Fig. 1E, step 3 and 4).

To record as many variations as possible within minutes, we used imaging rates of 1.9 stacks/s (each stack is 10 z-planes) for recordings under stimulation and imaging rates up to 7.2 stacks/s for activity from non-stimulated ORNs (spontaneous activity). The number of repetitions, so-called trials (Fig. 1E, step 3 and 4) was variable, depending on the chosen stimulus type and stimulus concentration. Since amino acids are the adequate stimuli for glomeruli from the lateral cluster, one of the 15 single amino acids or a mixture of them (AA) was used to stimulate those glomeruli. Trials under stimulations were alternated with trials without stimulation. A final stimulation was performed in order to confirm vitality of the tissue and the functionality of target glomeruli (Fig. 1E, step 5).

**Figure 2:**
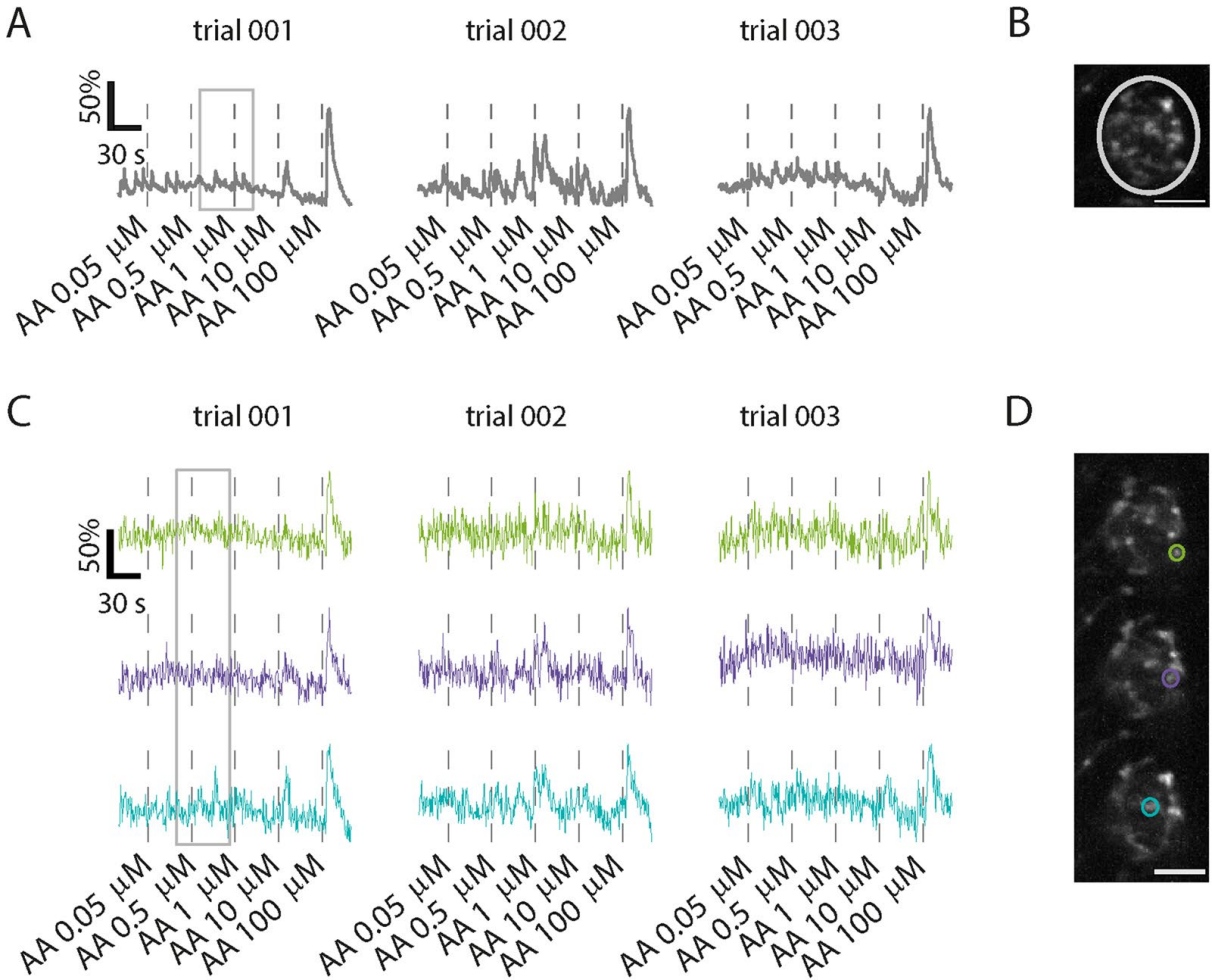
Calcium-related fluorescence over trials and between intraglomerular regions of interest. (**A**) Glomerular activity traces from the region of interest (ROI) shown in B. The following AA-concentrations are used: 0.05 µM, 0.5 µM, 1 µM, 10 µM and 100 µM. The traces reveal fluctuation over trials (imaging rate: 1.91 stacks/s). In this example, fluctuations are most apparent after AA concentrations in the lower micromolar range (< 10 µM). **(B**) Maximum projection in z and time of an exemplary glomerulus. The white circle represents a ROI that covers the whole glomerulus. Scale bar 10 µm. (**C**) Three consecutive trials under AA stimulation (imaging rate: 1.91 stacks/s). The colors used for the traces correspond to the colors of the ROIs shown in (**D**). The gray rectangle points out an exemplary time window in which variations between traces are observed. The cyan trace contains a peak within this time window that is mostly absent in the green and purple traces. (**D**) Three consecutive z-planes and intraglomerular example ROIs. Scale bar: 10 µm. *AA* a mixture of l-alanine, l-arginine, l-cysteine, l-glycine, l-histidine, l-isoleucine, l-leucine, l-lysine, l-methionine, l-phenylalanine, l-proline, l-serine, l-threonine, l-tryptophan, l-valine. Matlab and Adobe Illustrator were used for composition of this figure (see “Methods” for further details about the used software).

As hypothesized, trials under stimulations reveal partly similar and partly variable calcium fluctuations (Fig. 2). Under stimulations, glomerular averaged responses vary clearly over trials (Fig. 2A,B). Compared to the fluctuations over trials (Fig. 2A), the fluctuations among intraglomerular regions of interest are much smaller (Fig. 2C,D). Some of those regions, all having approximately the same size (Fig. 2D), contribute more to those fluctuations than others do (Fig. 2C). Furthermore, calcium variations are most pronounced after the application of lower-concentrated AA (< 10 µM, Fig. 2C).

**Figure 3:**
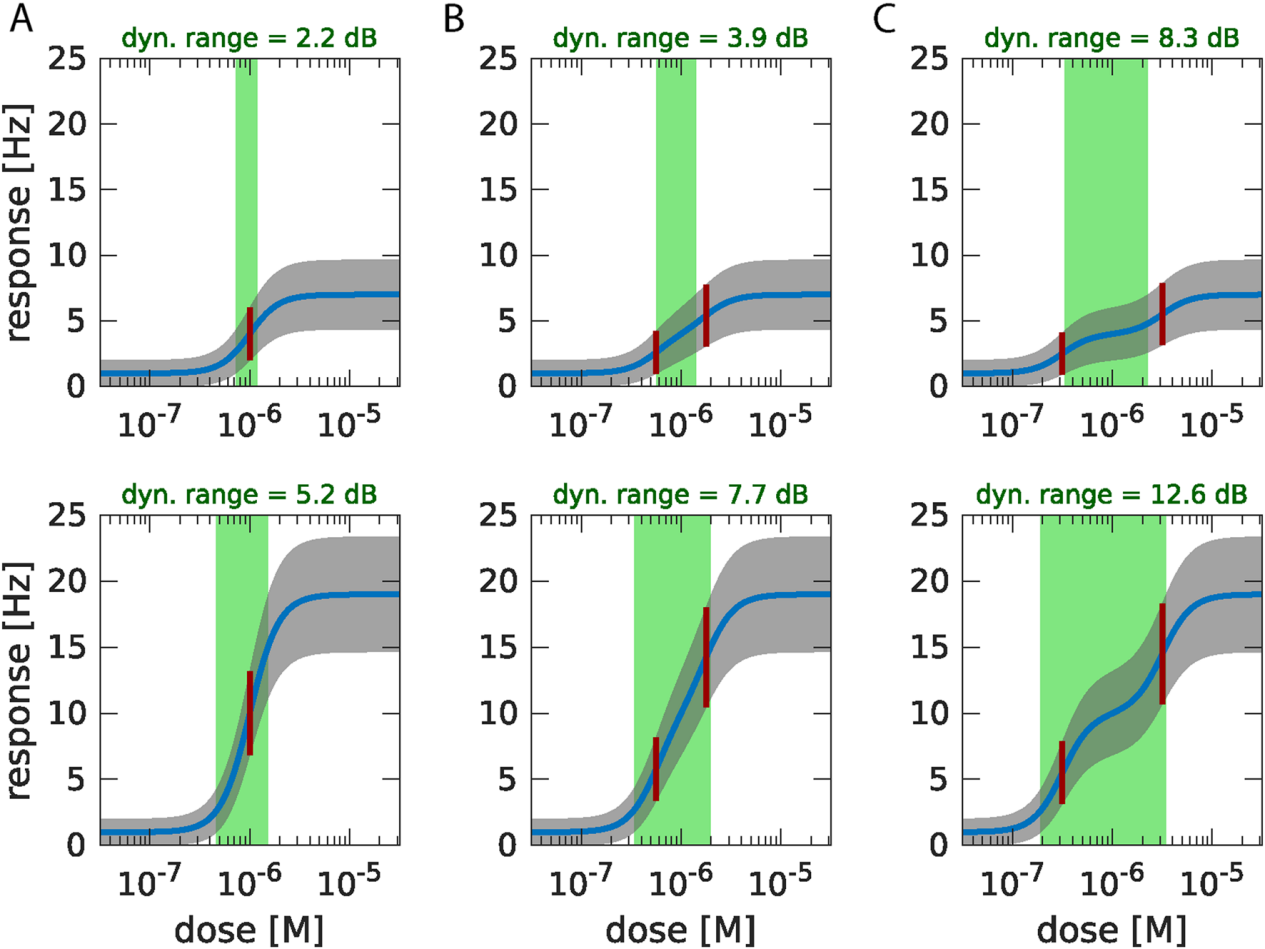
Effect of heterogeneous ORN sensitivity distribution on input dynamic range. Odour dose–response curves (blue lines) are simulated for exemplary ORN ensembles with different sensitivities to odour concentration and maximum response AP frequencies. The trial-to-trial standard deviation (*SD*) of the ensemble response frequency is shown in grey. The input dynamic range (*DR*, stimulus range with responses that are significantly different from minimum and maximum) is shown as green area and quantified in dB between its minimum and maximum dose. (**A**) Simple configuration consisting of a single ORN group with homogeneous sensitivity (*CD50* = 10^–6^ M, see position of red line, also showing corresponding response frequency ± *SD*), a combined basal AP frequency of *f*_0 _= 1 Hz, and a combined maximum frequency of *f*_max_ = 7 Hz (top) or 19 Hz (bottom). For larger *f*_max_, *DR* increases. (**B**) Two ORN groups with *f*_max_ as in A, but with distinct sensitivities (*CD*50 = 10^–6.25^ M and 10^–5.75^ M, red lines). *DR* are increased compared to (**A**). (**C**) Same as (**B**), with sensitivities further apart (*CD50* = 10^–6.5^ M and 10^–5.5^ M). While *DR* are further increased compared to B, the dose–response curves begin to plateau between the two *CD50* concentrations instead of increasing linearly, because they are too far apart. Dose–response curves for each sensitivity group are modelled with a Hill equation (Hill coefficient of 3) and summed. Trial-to-trial *SD* at response frequency f is calculated for a measurement of 1 s with the assumption of Poisson-distributed AP counts, giving $SD = \sqrt f$*.* Input dynamic range is defined as the range of stimulus concentrations [*c*_*min*_*, c*_*max*_] that satisfy *f*(*c*_*min*_) *− SD*(*c*_*min*_) > *f*_*0*_ and *f*(*c*_*max*_) + *SD*(*c*_*max*_) < *f*_*max*_*. DR* = 10 × log_10_(c_max_/c_min_) dB is used for quantification of the input dynamic range. Matlab and Adobe Illustrator were used for composition of this figure (see “Methods” for further details about the used software).

The grey rectangle shown in Fig. 2C, trial 1, is an exemplary time window and points out a calcium peak in the blue lower trace which is absent in the other traces (Fig. 2C). These calcium fluctuations among intraglomerular ROIs might reflect the multidimensional ORN activity of ORN ensembles, observed at the level of the epithelium. For example, ORNs could have different sensitivities, which would improve their overall dynamic range (explained in simulations shown Fig. 3). In case the calcium fluctuations observed in glomeruli (Fig. 2C) indeed reflect this multidimensional activity, similar calcium peaks should be present in all other small ROIs that cover the same axon and hence, the activity of those ROIs will be correlated throughout the axon terminal. Moreover, such ORN-specific peaks should be absent in ROIs that cover other axons. On the other hand, the fluctuations could also represent activity that is local, for example, calcium fluxes that occur after the spontaneous opening of calcium channels in axon terminals or signaling from intracellular calcium stores. Peaks should then be restricted to one or a few nearby ROIs (no correlation throughout the corresponding axon terminal). Accordingly, mapping correlated activity could serve as an indication for the type of activity we are measuring, either ORN-specific activity or local activity.

### Correlation-based selection of intraglomerular regions of interest

**Figure 4:**
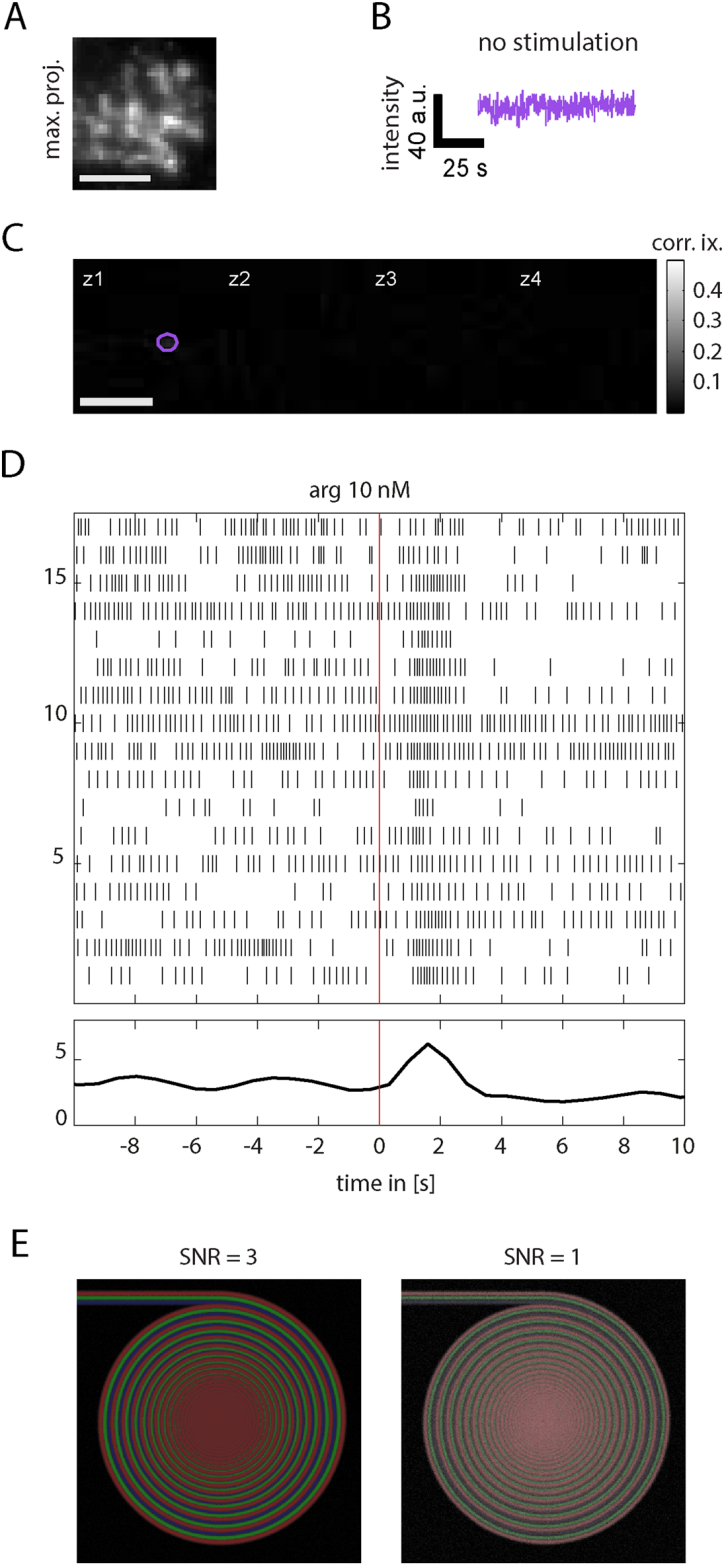
Exemplary activity measurements from glomerular calcium imaging and ORN cell-attached patch-clamp recordings and their implications on ROI correlation. (**A**) Presynaptic calcium imaging of a whole glomerulus, maximum-projected in time. (**B**) ROI time trace from recording without stimulation. (**C**) Activity correlation imaging (ACI) from trace in B results in no usable correlation map. (**D**) Repeated ORN on-cell patch-clamp recordings during single, low-intensity (10 nM l-arginine) olfactory stimulus at t = 0 s. Each detected action potential is shown as vertical line for each trial. Only the firing rate from the combined trials (lower plot) exhibits a significant increase upon stimulation, while individual trials are partly indistinguishable from spontaneous firing. (**E**) Component separation ability in ACI maps depending on signal to noise ratio (SNR) and spatial distance. Spiking calcium activity is simulated for three components and spatially distributed along three 2D converging spirals. Each spiral has a gaussian line radius of σ = 3 pixels and a maximum spatial separation of 6 pixels (outside) and minimum 0 pixels (at the center). Activity correlation images are calculated (with component colors red, green and blue) for a SRN of 3 (left) and 1 (right). Reliable pixel component identification is possible for signals at σ/2 spatial overlap for SNR 1 and even smaller for SNR 3. Matlab and Adobe Illustrator were used for composition of this figure (see “Methods” for further details about the used software).

An image of color-coded correlated activities will enable the visualization of regions with distinct activity. This analytical procedure—called activity correlation imaging (ACI)—was previously used for the distinction of mitral cell activities^22^. We investigated the extent of ACI applicability for intraglomerular calcium traces from ORN axon terminals. Previously, for mitral cells, an optimal separation was achieved during spontaneous activity^22^. Therefore, we performed an initial ACI analysis using no-stimulation trials (spontaneous activity) from small intraglomerular ROIs first. Our data show that typical calcium traces from non-stimulated ORNs on the level of the olfactory bulb scarcely show distinct events and thus making a correlation-based analysis unsuitable per se (Fig. 4A–C). A set of electrophysiological experiments from ORNs on the level of the epithelium reveal that amino-acid responsive ORNs from *Xenopus laevis* tadpoles do fire spontaneously with firing rates of multiple spikes per second (Fig. 4D, firing before stimulation).

As the next step, we simulated noisy calcium traces that resemble recorded stimulus-induced calcium traces to study the extent of ACI applicability for our assay in general. Traces are called identical in this context when they were generated from identical spike times (even if individual pixels have different photon shot noise). Traces are called distinct when generated from different spike times, although they can become similar to each other when spontaneous events are rare and responses to the simulated stimuli are similar. Spike times are poisson-distributed based on the assumed stimulus response frequency.

Identical and distinct calcium traces were used in two ways. Firstly, to test the spatial separation capability of the ACI analysis, image series were generated for a combination of three distinct calcium traces, with each trace being positioned on separate 2D spiral pathways that converge towards the center (Fig. 4E). Reducing the signal-to-noise ratio of these traces from three [SNR = 3, Fig. 4E (left)] to one [SNR = 1, Fig. 4E (right)], resulted in a reduced spatial separation among densely packed axons (centers of the images).

**Figure 5:**
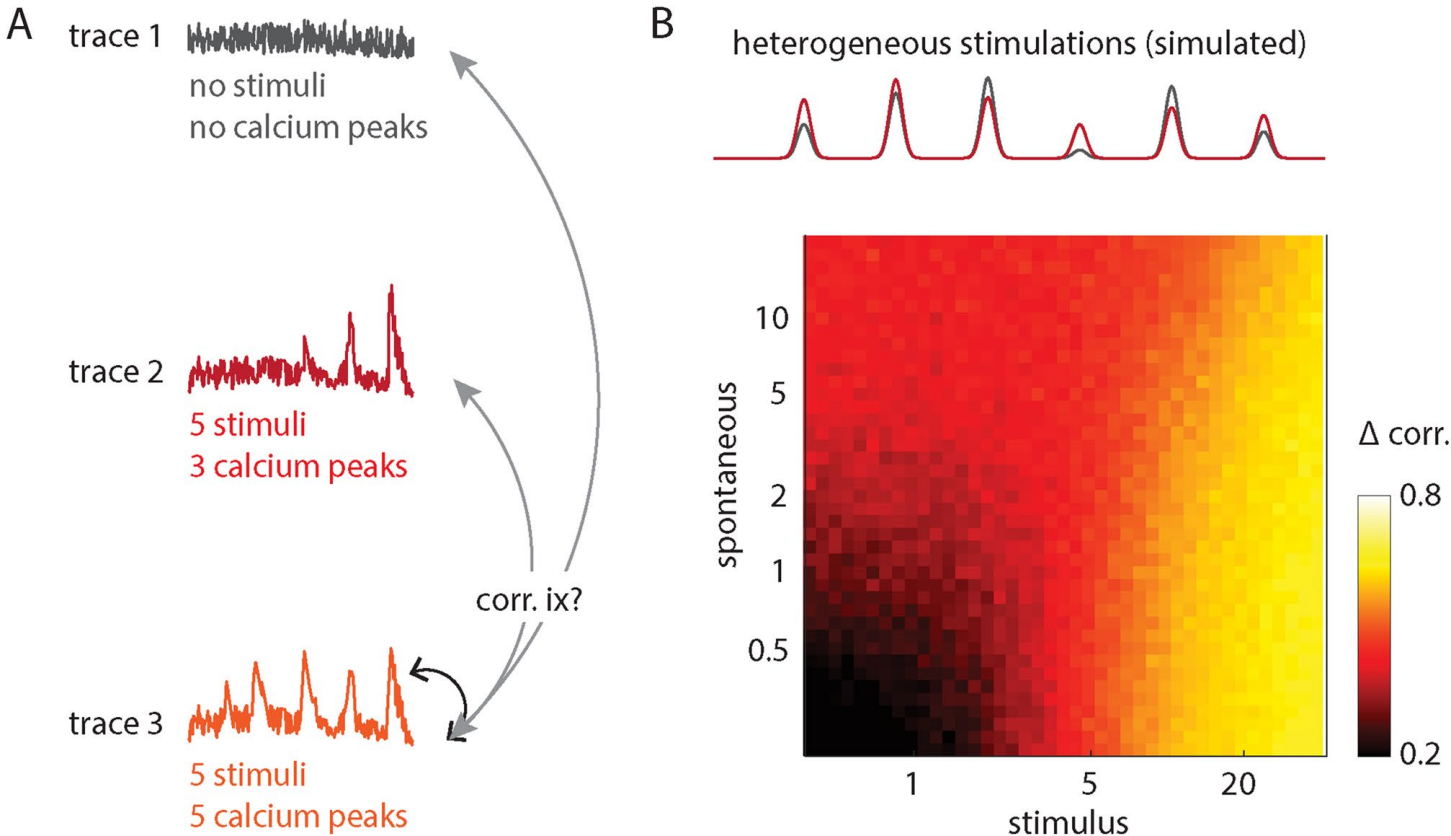
Calcium correlation dependence on stimulated and spontaneous activity. (**A**) Example of three ROI Ca^2+^ traces (indices ix) acquired under different stimulus conditions: control (no stimulus) and two stimulus strengths, resulting in a different number of detectable Ca^2+^ peaks. Inter-trace correlation coefficients depend on spontaneous and stimulus-induced calcium activity. (**B**) Simulation of trace correlation coefficients. Stimulated and spontaneous firing rate was simulated for different ROIs with each ROI being heterogeneously stimulated at 6 different stimulation times (two example ROI stimulation amplitudes shown in plot). For each ROI, 20 Calcium traces were simulated (for details see Supplementary Fig. S2) and pairwise trace correlation coefficients were calculated. Correlations between traces belonging to the same ROI (“intra-ROI”) are usually higher than correlations between traces from different ROIs (“inter-ROI”). The correlation difference (“Δ corr.”) is shown color-coded in the picture, with each pixel representing the difference between average correlations of intra-ROI and inter-ROI traces, and pixel positions indicating the parameters used for each simulation set (spontaneous firing rate and stimulation strength). Matlab and Adobe Illustrator were used for composition of this figure (see “Methods” for further details about the used software).

Secondly, identical and distinct calcium traces—differing in their number of spontaneous events, on the one hand, and differing in the amplitude of stimulus-induced responses, on the other—were used to investigate how the number of events influences correlation coefficients and a subsequent ACI-based separation. The difference between stimulus-induced correlation among identical calcium traces [Fig. 5A (black arrow)] and correlation between distinct calcium traces [Fig. 5A (grey arrows)] represents a correlation “contrast” that enables visualization of distinguishable activity (Fig. 5B, Supplementary Fig. S1). Calcium events induced by heterogeneous stimulations (Fig. 5B) appear to be preferred over calcium events induced by homogeneous stimulations, as they evoke higher correlation contrast (for technical details see Supplementary Fig. S1D).

**Figure 6:**
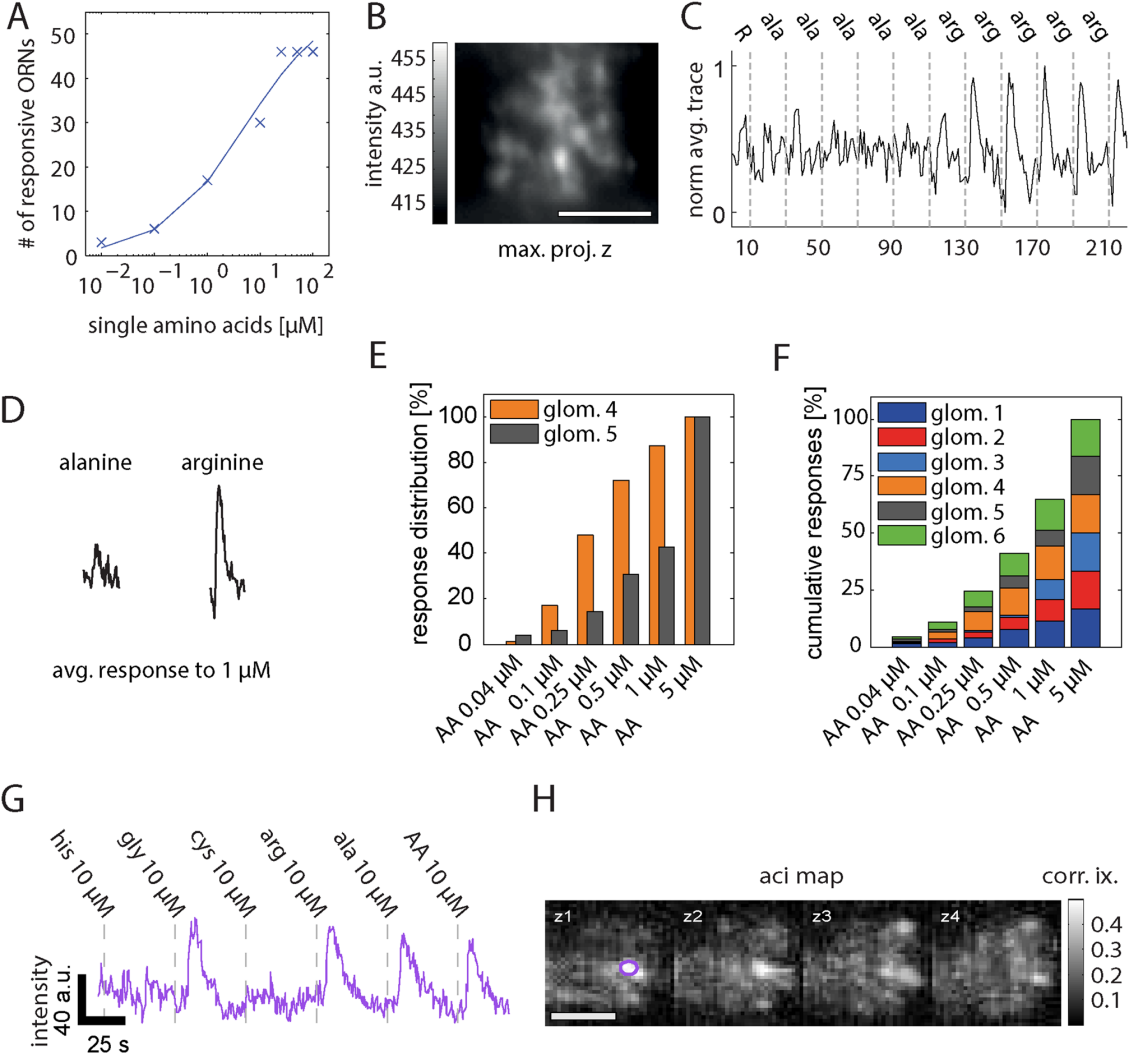
Amino-acid-induced ORN response profiles and dynamic ranges. (**A**) Number of ORNs that respond to the corresponding concentration of single amino acids. EC50-value is calculated to be 8.8 μM (patch clamp data from olfactory epithelium). (**B**–**H**) calcium-imaging data from the olfactory bulb. (**B**) Maximum projection of a glomerulus stimulated with single amino acids. (**C**) Response trace averaged for all z-planes normalized to the maximum response. (**D**) Typical response to 1 µM l-alanine and 1 µM l-arginine (average from all applications of this experiment). X- and y-scale as in (**C**). (**E**) Percentage of ROIs of glomerulus 4 and 5 that respond to six different AA concentrations. For both glomeruli all ROIs respond to 5 μM AA. (**F**) Similar to (**E**), now showing the cumulative percentages for all glomeruli (n = 6) measured within this condition. (**G**) For measuring stimulus-induced responses suitable for activity correlation imaging, fast 4D time series were recorded using imaging rates of 1.9 stacks/s. Responses are induced with the application of 10 μM l-glycine, l-arginine and l-alanine. An example raw intensity trace from such a recording is shown in purple. The raw intensity trace shows the averaged activity (in x and y) from a small region of interest drawn in one of the recorded z-planes (purple circle, plane z1). (**H**) Correlation is elevated widespread throughout the glomerulus. Matlab and Adobe Illustrator were used for composition of this figure (see “Methods” for further details about the used software).

Accordingly, we searched for physiological stimuli that possibly induce heterogeneous responses, by obtaining complementary electrophysiological and calcium-imaging data. We focused on the application of different amino acid concentrations, either using single amino acids or a mixture of them (Fig. 6). We also explored the dynamic range of amino-acid responsive ORNs at the level of the olfactory epithelium and at the level of the olfactory bulb (Fig. 6). At the level of the olfactory epithelium the EC-50 value of ORNs was calculated to be 8.8 µM using single amino acids (Fig. 6A). At the level of the olfactory bulb, three out of five glomeruli responded to concentrations of 1 µM (single amino acids, example shown in Fig. 6B,C), although response maxima were variable (Fig. 6D, Supplementary Fig. S2). When using an amino-acid mixture instead, more than 50% of the intraglomerular ROIs responded to concentrations of 1 µM and all ROIs were responsive to an amino-acid mixture concentration of 5 µM (Fig. 6E,F). Stimulus-induced responses using the same concentration resulted in homogeneous correlation throughout a glomerulus (Fig. 6G,H).

**Figure 7:**
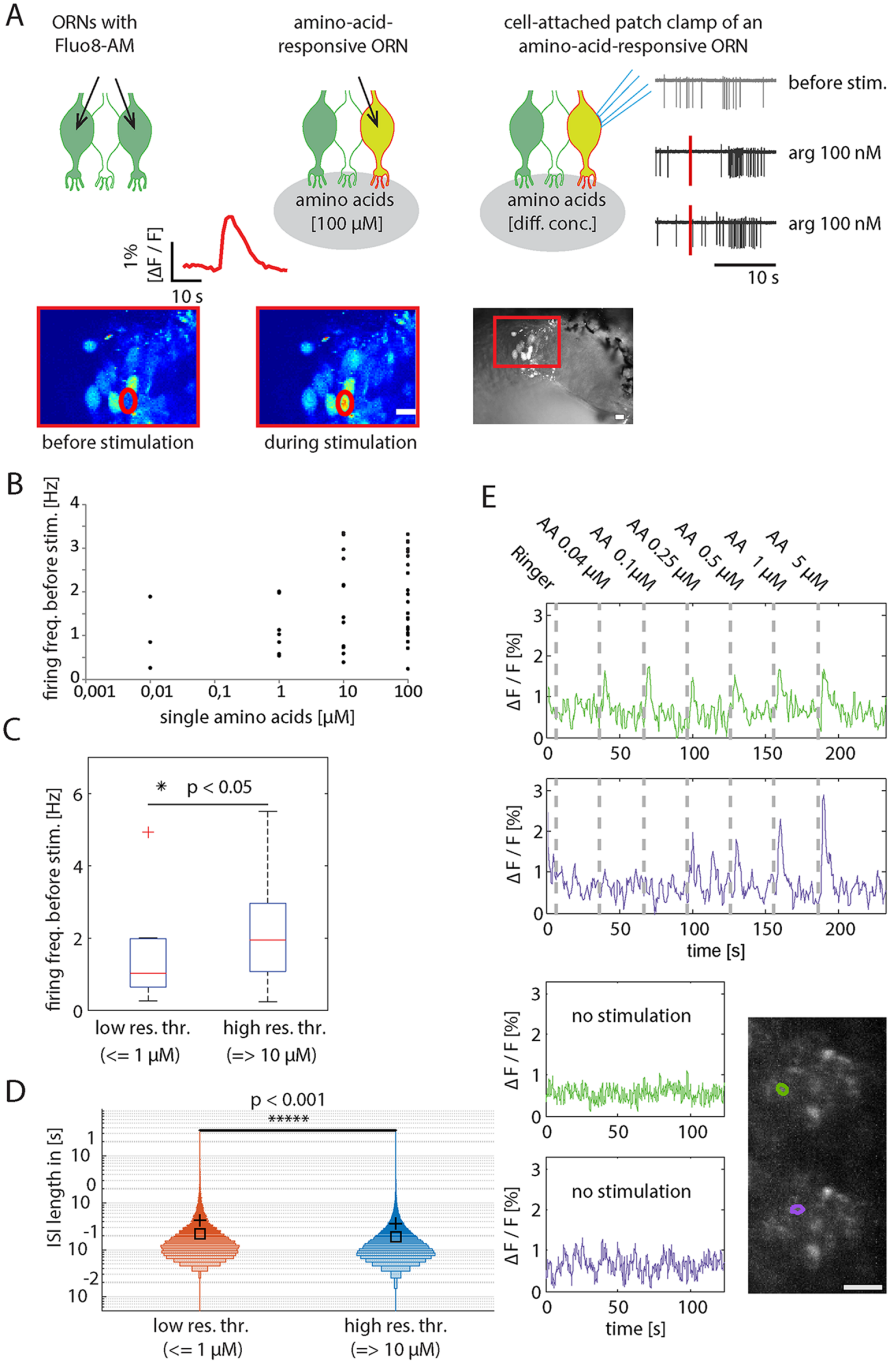
Heterogeneity found among activity patterns of amino-acid responsive ORNs, indicate distinct dynamic ranges within individual glomeruli. (**A**) Selection of amino-acid-responsive ORNs for cell-attached patch clamp recordings. At the level of the epithelium, ORNs are temporarily stained with the calcium indicator Fluo8-AM (left). Similar to the calcium-imaging recordings from glomeruli, amino acids are administered just before the olfactory epithelium (middle). Calcium imaging of ORN somata is performed to locate amino-acid-responsive ORNs (red circle, left and middle). A bright field image is used to guide the patch-pipette to one of the amino-acid-responsive ORNs (right image). Exemplary patch-clamp recordings (traces on the right), showing an example of spontaneous activity and two examples of an l-arginine-induced response in the same ORN. (**B**) ORNs (single dots) sorted based on their response thresholds to single amino acids [l-arginine, l-histidine, l-lysine, l-methionine, l-leucine, l-alanine, l-tryptophan, or l-phenylalanine, (x-axis)] and their spontaneous firing rates (y-axis). EC-50 was calculated to be 8.8 µM. (**C**) Using the EC-50 value (8.8 µM) for testing a relationship between ORNs sensitivity and their firing rates (Mann–Whitney-U-test: p = 0.016). ORNs with a low response threshold (n = 11) are responsive to amino acid concentrations of <  = 1 µM. ORNs with a high response threshold (n = 35) are responsive to amino acid concentrations of =  > 10 µM. (**D**) Distribution of ISI length of ORNs 30 s prior to the application of either stimulus solution to stimulation with either single amino acids (l-histidine, l-arginine, l-methionine) or Ringer’s solution (negative control). 9 ORNs with had a low response threshold (n = 13,440 ISIs; first response to stimulus concentrations below 1 of <  = 1 µM) and 49 ORNs had a high response threshold (n = 48,711 ISIs; first response to stimulus concentrations above 1 µM). The black cross displays the mean. The median is depicted as a black square. The histogram width is normalized to the maximum bin count of the belonging distribution. The bin size is 0.01 s. The y-axis is logarithmic and displays the ISI length in seconds [s]. The x-axis is categorical. p < 0.001 using the Wilcoxon rank sum test. (**E**) Example intraglomerular regions (left, in green and magenta) with distinct dynamic ranges (middle) and spontaneous activities (right). All scale bars: 10 µm. *AA* a mixture of l-alanine, l-arginine, l-cysteine, glycine, l-histidine, l-isoleucine, l-leucine, l-lysine, l-methionine, l-phenylalanine, l-proline, l-serine, l-threonine, l-tryptophan, l-valine. Matlab and Adobe Illustrator were used for composition of this figure (see “Methods” for further details about the used software).

During our search for physiological amino-acid concentrations using the patch-clamp technique (scheme of procedure in Fig. 7A), we observed higher spontaneous firing in some of the patched ORNs having a response threshold higher than 1 µM (Fig. 7B). When using the EC-50 value (8.8 µM) for categorizing this data in two groups, we got ORNs responding to amino-acid concentrations of < = 1 µM (low response threshold) and ORNs responding to amino-acid concentrations of = > 10 µM (high response threshold). Firing frequencies before stimulation differ significantly between groups (Fig. 7C, Mann–Whitney *U* test, p = 0.016). In a complementary experiment, using the same categorization criteria, we compared the inter-spike interval distribution of those groups, supporting this observation (Fig. 7D, Wilcoxon test: p < 0.001).

In addition, rapid 4D calcium-imaging recordings seem to be in line with this observation (Fig. 7E). Therefore, we quantified and compared calcium transients from glomeruli under stimulation or not and compared intensities ratios (maximum/median). No correlation was observed (Suppl. Fig. S3).

**Figure 8:**
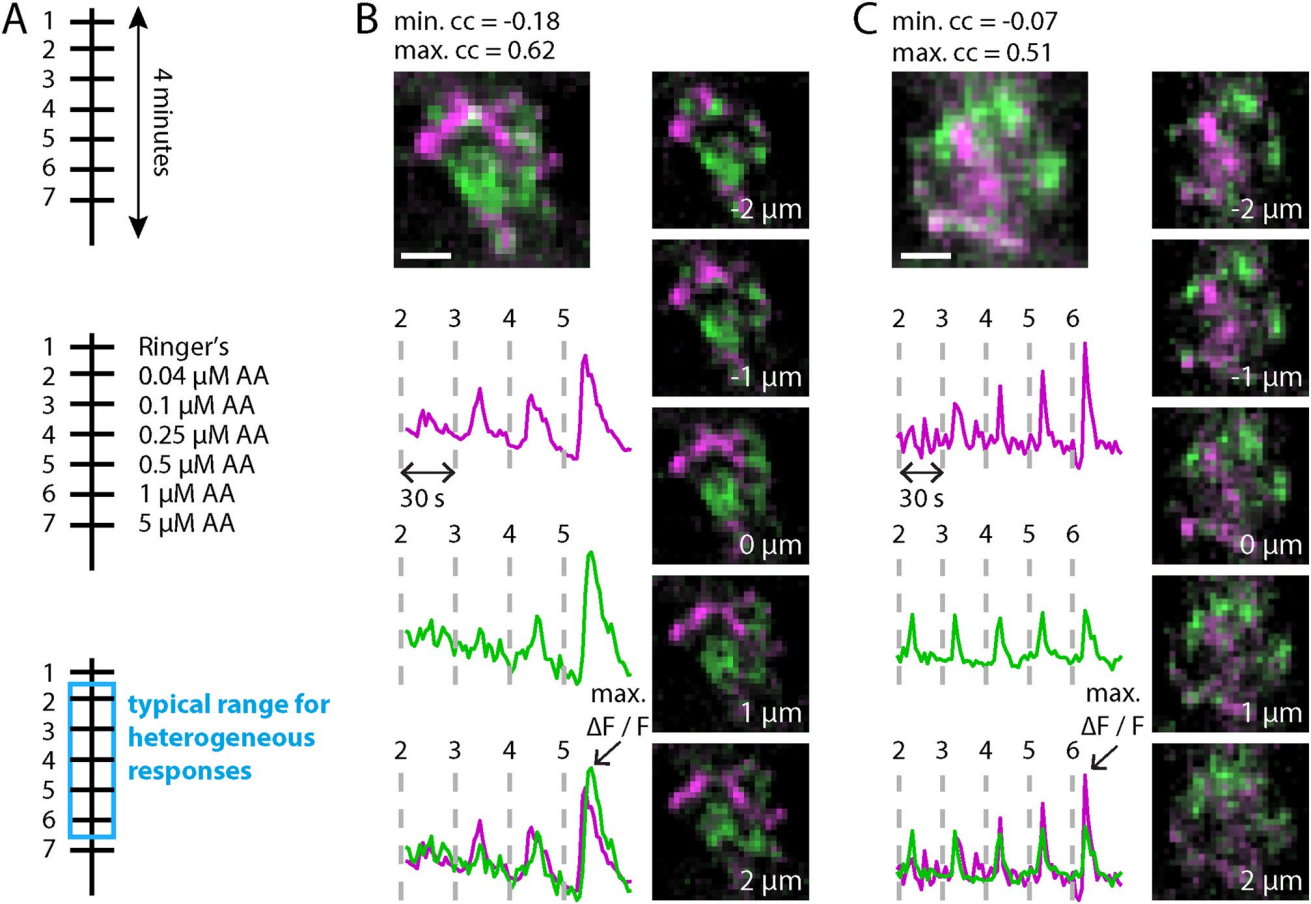
Glomeruli typically have two distinctive sensitivity profiles. (**A**) Timeline of an calcium-imaging recording. A typical calcium-imaging recording takes around four minutes. During a recording seven or eight stimuli with different concentrations are applied (interstimulus interval: 30 s). The concentrations used cover the dynamic range of a glomerulus (see also Supplementary Fig. S5). Heterogeneous responses are typically induced by concentrations of 0.04–1 µM. (**B**,**C**) Two example glomeruli with two distinctive sensitivity profiles. Up left: color projection in z of the two correlation maps in magenta and green, respectively. The two corresponding reference traces [normalized to the maximum response (max ΔF/F, arrows)] are shown individually and as an overlay, below the color projection. The AA concentrations used lie in the typical range for heterogeneous responses, shown in (**A**). To finish up, five single z-planes—third plane is center of glomerulus—are shown revealing the projections of the axon terminals with distinct sensitivities in details. Scale bars: 5 µm. *AA* a mixture of l-alanine, l-arginine, l-cysteine, glycine, l-histidine, l-isoleucine, l-leucine, l-lysine, l-methionine, l-phenylalanine, l-proline, l-serine, l-threonine, l-tryptophan, l-valine. Matlab and Adobe Illustrator were used for composition of this figure (see “Methods” for further details about the used software).

Up to this point, intraglomerular ROIs were drawn and selected manually. In order to reduce ROI selection biases, we automatized the imaging processing steps, including the ROI selection procedure and the corresponding correlation maps (see “Methods”). This analysis was applied on 11 recordings, revealing two components, except for one glomerulus (Supplementary Fig. S4). Typically, heterogeneous responses within individual glomeruli were induced by odor concentrations of 1 µM or lower (Fig. 8). As a control analysis, we mapped the degree of response fluctuations to intraglomerular regions of distinct intensities. No significant differences were observed (Suppl. Fig. S5).

Based on these observations, we conclude that olfactory glomeruli from *Xenopus laevis* tadpoles (developmental stages 51–54), typically contain two sensitive components with distinct dynamic ranges (Fig. 8).

As a last step, we estimated the number of functional axons that actually enter a glomerulus. Starting from the typical size of a glomerulus for our species, we estimate the maximal number of presynaptic axons that enter a glomerulus to be about $43~ \pm 18$ for *Xenopus laevis* tadpoles (stages 51–54; compared to 1000–2000 in mice from convergence ratios, Ressler et al., 1994), according to the following assumptions.

The available volume of a glomerulus is approximated by its typical radius $r_{g}$ and assumed to be fully occupied by the volumes of ORN axons $v_{a}$, mitral cell dendrites $v_{d}$ and the volume of synapse pairs $v_{s}$ for each pair of ORN and mitral cells:

1 $$\frac{4}{3}\pi r_{g}^{3} = n_{a} v_{a} + n_{d} v_{d} + n_{a} n_{d} v_{s}$$

Rewriting their respective numbers ($n_{a}$, $n_{d}$) in terms of the ratio $R_{a} = n_{a} /n$ to the total number of processes $n~ = ~(n_{a} + n_{d} )$, and setting the volume of both processes (axons and dendrites) to $v_{a} = v_{d} = \pi r_{a}^{2} lb$, with $r_{a}$ being the radius and $l$ the length of one branch for $b$ branches for each process in the glomerulus, the equation above becomes

2 $$\frac{4}{3}\pi r_{g}^{3} = b~l~\pi r_{a}^{2} ~n + R_{a} ~(1 - R_{a} )~v_{s} n^{2}$$

Solving for $n$ and using parameters taken from literature and our own observations (*Xenopus laevis* tadpoles, stages 51–54): $r_{g} = 7.5 \pm 1$ μm (glomerular radius, based on^23^ and own results), $b = 4 \pm 1$ (number of axonal branches, based on^23,24^), $l = 50 \pm 20$ μm and, $r_{a} = 0.18~ \pm 0.02$ μm (length and radius of axonal branch, based on olfactory nerve^25^), $R_{a} = 5/6$ (ratio, based on^5^) $v_{s} = 2 \pm 0.5$ μm^3^ (synapse pairs, estimated based on ^5^), then gives a total number of $n = 51 \pm 21$ processes, resulting in the number of presynaptic processes for tadpoles from *Xenopus laevis* (stages 51–54).

$$n_{a} = nR_{a} = ~43~ \pm 18$$.

Based on these estimations, we conclude that (1) within glomeruli there are heterogeneous activity patterns (Fig. 8, Supplementary Fig. S4) and (2) this heterogeneity is so pronounced that we are able to separate those activities with a correlation-based analysis. Furthermore, since we estimate $43~ \pm 18$ ORN axons innervating a typical glomerulus in tadpoles, these activities probably reflect the activity of two axonal subgroups, but not single ORNs.

## Discussion

**Figure 9:**
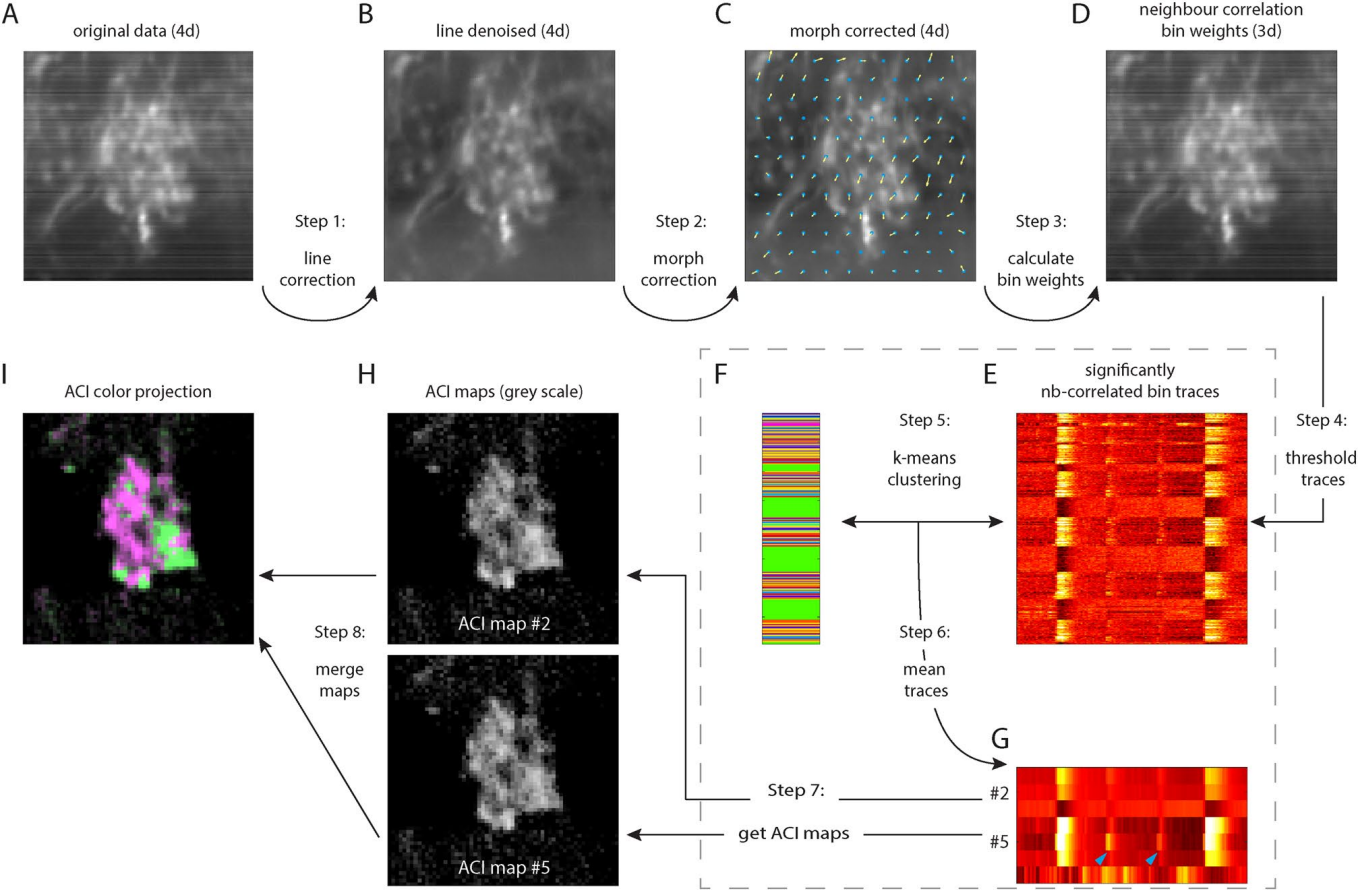
ROI autodetection scheme. (**A**) 4D imaged data (time series of 3D stacks, shown here as mean-projection in time for one example z-layer) contains CCD detector offset artifacts. (**B**) Line artifacts are reduced by subtraction of averaged line signal. (**C**) Image drift and tissue distortion over time are reduced by sequentially applying shift compensation, optical flow field detection and weighted de-morphing. (**D**) Regions containing significant calcium activity have higher correlation between each center and surrounding pixel (“neighborhood (nb)-correlation”). The average nb-correlation per pixel bin is shown here for one example z-layer. (**E**–**G**) Trace selection procedure. (**E**) Average traces for each bin are calculated using weighted averaging (pixel weight = nb-correlation), and only traces with significantly elevated correlation (one standard deviation above average) are collected. Selected and normalized calcium traces are shown in picture with time in x dimension and trace index in y dimension. (**F**) Clusters of similar traces are obtained using k-means clustering of trace data. Color-coded cluster assignment is shown for each trace in image. (**G**) all traces belonging to the same cluster are averaged. Two of the 7 averaged traces are selected for the following example. (**H**) Each pixel of the 4D data is correlated in time with the selected traces, producing 3D activity correlation maps (ACI-maps). (**I**) Color projection of ACI maps. The ACI maps are merged by assigning a color for each map and choosing pixel color and intensity by highest-correlated map color and correlation value, respectively. Matlab and Adobe Illustrator were used for composition of this figure (see “Methods” for further details about the used software).

Intraglomerular axons have been visualized before, either anatomically using single cell electroporation (*Xenopus laevis*^26^) or functionally by utilizing altered levels of the olfactory marker protein (in mice^13^). Here, we demonstrate how to use calcium fluctuations for the visualization of intraglomerular axon terminals with distinct dynamic ranges (Figs. 8, 9). To achieve visual separation, a sufficiently variable stimulus protocol, fast 4D calcium imaging as well as correlation imaging is necessary.

ORNs have, in addition to odor-induced activity, significant stimulus-independent (spontaneous) activity, which is physiologically important^20^. How spontaneous activity is related to ORN sensitivity in *Xenopus laevis* is beyond the scope of this study, although in mice^27^ and *Drosophila*^28^ spontaneous activity plays a crucial role in odor dynamics. This seems to be the case for *Xenopus laevis* as well, since our patch-clamp recordings indicate an inverse relationship between the odor-response thresholds and the basal firing rates of amino-acid responsive ORNs (Fig. 7). Nonetheless, the observation that out of 46 ORNs only 3 were sensitive to low concentrations does not imply there are no ORNs exhibiting high spontaneous firing rates and being responsive to odor-concentrations of < = 1 µM.

The firing rates before stimulation obtained using the cell-attached mode are consistent with the rate of 1–4 spikes/s reported for different other species^14,18,20,29–31^. Furthermore, we found that ORNs of *Xenopus laevis* tadpoles respond to application of single amino acids in a wide concentration range. Three out of 46 neurons (Fig. 4B) responded to stimulus concentrations as low as 10 nM, whereas all tested ORNs responded to amino acid concentrations as high as 50 µM. We obtained an EC-50 value of 8.8 µM (Fig. 6), which is in line with previously reported EC-values in other species: 3–90 μM in tiger salamander^32^, 4.4–104 μM in mice^19^ and 1–10 μM in rats^33^.

An additional observation from our patch-clamp experiments was the high variability among the recordings using low concentrated amino acids as stimuli. Aligning successive recordings from the same neuron suggests on the one hand that ORNs indeed react to such low odor concentrations (Fig. 4). On the other hand, it indicates correlation or integration as a possible way to distinguish between spontaneous activity and odor-induced activity that are otherwise difficult to discriminate on the level of single ORNs. Moreover, such computation in ORNs might be crucial for the regularly observed glomerular responses to odors in the nanomolar range (Fig. 3). Interestingly, correlated odor responses turned out to be required for the successful visualization of intraglomerular axons using ACI (Fig. 5, Supplementary Fig. S1).

Previously, spontaneous activity of mitral cells was used for discrimination of mitral cell networks^22^ using ACI. In our preparation aiming for the separation of ORN axons, however, this approach was not successful. Methodological differences may explain why calcium imaging of spontaneous activity was not suitable for presynaptic axon terminals within glomeruli. Firstly, for our preparation we used CaGreen (K_*d*_ 190 nM), a calcium sensor that is suitable for electroporation, instead of Fluo-4/AM (K_*d*_ 345 nM), which is a membrane permeable calcium sensor used for ACI before^22^, but not suitable for staining the presynaptic side of glomeruli. Secondly, individual action potentials might not translate into detectable calcium increase at presynaptic compartments. Thirdly, despite imaging rates of up to 72 frames/s, our time resolution is not in the range of single action potentials at which larger differences are expected.

Taken together, the glomerular morphology and the use of CaGreen limit the application of ACI as shown in Figs. 4 and 6G,H. However, the used parameters in this study is currently the best compromise between spatial and temporal resolutions. Above all, the listed limitations neither affect our observation that a glomerulus contains heterogeneous responses (Supplementary Fig. S5) nor our conclusion that intraglomerular activity is correlated but distinguishable (Supplementary Fig. S4). Yet, functional super resolution microscopy—obviously in combination with calcium sensors that are suitable for super resolution microscopy—would overcome these limitations resolution and would therefore be an ideal validation for our findings. Recently, functional super resolution microscopy has been applied for recordings of short calcium trials^34^. However, for this method in vivo applications are typically limited to a few images taken at large intervals^35,36^. Accordingly, to the best of our knowledge, it is not yet applicable for long recordings (multiple minutes) like ours.

In contrast to spontaneous activity, odor-induced activity was easily detected in ORN axon terminals using calcium imaging. Our data revealed that, in general, the stimulation-dependent correlation of the signals were prohibitively large and widespread for axonal differentiation. Yet, we observed that intraglomerular regions differed in odor-response thresholds and in the range of odor concentrations to which their responses change (dynamic range). We therefore utilized these properties by devising a stimulation protocol, covering a range of concentrations. When using nanomolar to lower micromolar odor concentrations, it was possible to reduce inter-axonal correlation so that axons could be visually discriminated using ACI (Fig. 8, Supplementary Fig. S4). Activity patterns of the discriminated projections differed in sensitivity, which possibly broadens the dynamic range of glomeruli (Fig. 3).

To improve signal quality and to obtain broad dynamic ranges within a glomerulus, it would be beneficial for each glomerulus to have many terminating ORN axons with distinct sensitivities. Therefore, since we distinguished two activity patterns per glomerulus only, we consider our observations as the minimal number of ORNs and its axon branches that project to the same glomerulus.

There are several explanations for the low number of detected activity patterns. At first, stimulus-induced correlation throughout a glomerulus interferes with the axonal distinction, possibly leading to false negatives (less axons detected than present). Secondly, of all the ORNs that terminate in one glomerulus many ORNs might not have been electroporated. The latter is highly probable, since many electroporation-related parameters are prone to variations, especially the positioning of the electrodes (for a detailed protocol for ORN electroporation see^37^). Calculating the number of ORNs per glomerulus would provide more insight into the actual number of ORNs that are not stained by electroporation. A combination of electroporation and a retrograde ORN staining through a bolus injection^38^ would be a suitable approach to obtain these numbers. However, this approach would induce damage to the tissue and would render the resulting preparation incomparable to the undisturbed one we employed in this study. Thirdly, the use of an amino-acid mixture^39^ or the continuously changing ORN sensitivity^40^ could interfere with the detected number of components. Also, Manzini and Schild showed that ORNs have specific amino-acid response profiles^15^. Accordingly, successive stimulation of ORNs with combinations of individual amino acids at different concentrations might reveal additional functional activity patterns at the presynaptic side of glomeruli. However, experimental limitations such as bleaching, morphological changes over time and tissue vitality, made this approach unfeasible. The above reasoning supports the common assumption that there are multiple axons converging into a glomerulus. Taken together, we expect typically two groups of functionally distinguishable axons that terminate in a single glomerulus (Fig. 8). Those patterns differ in odor-response thresholds and dynamic range. In conclusion, we presented a method that enables the differentiation of distinct intraglomerular activity patterns and their corresponding axonal projections. From now on, one can thus study how these activity patterns contribute to the glomerular computation of presynaptic stimulus-induced input. While our work was performed on *Xenopus* *laevis* this method is generally applicable to neuropils across species if their input is tunable.

## Methods

### Animals and stimuli

Experiments were performed according to the regulations of the Ethics Committee of the University Medical Center Göttingen, Germany, and to the regulations of the relevant Ethics Committee of the State Authority (the LAVES Office of Lower Saxony, Germany). Experimental procedures, specifically for electroporation (with biological markers) of *Xenopus laevis* larvae and sacrificing them, were approved by the Animal Welfare Committee of the University Medical Center Göttingen (Tierschutzkommission, Universitätsmedizin Göttingen, 37099 Göttingen). Experiments and data analysis procedures were performed in compliance with the ARRIVE guidelines (https://arriveguidelines.org).

All data were obtained from olfactory receptor neurons of *Xenopus laevis* tadpoles (of either sex, stage 51–54)^41^. To stimulate the neurons we used fifteen single amino acids dissolved in Ringer’s solution: l-alanine, l-arginine, l-cysteine, glycine, l-histidine, l-isoleucine, l-leucine, l-lysine, l-methionine, l-phenylalanine, l-proline, l-serine, l-threonine, l-tryptophan, l-valine (Sigma-Aldrich) or a mixture of them^42^. The composition of Ringer's solution was: 98 mM NaCl, 2 mM KCl, 1 mM CaCl_2_, 2 mM MgCl_2_, 5 mM glucose, 5 mM sodium pyruvate, 10 mM HEPES. Osmolarity was adjusted to 225–235 mOsm/l and pH to 7.8.

### Sample preparation for patch-clamp recordings at the level of the olfactory epithelium

*Xenopus laevis* tadpoles were anesthetized with ice water. After 3 min, animals were sacrificed through a transection between the brain stem and spinal cord. Tissue samples included the olfactory epithelium, the olfactory nerve and the anterior part of the brain. For cell-attached patch-clamp recordings, the sample tissue block was glued onto a specimen holder and placed into a vibroslicer stage (VT 1200S; Leica Microsystems GmbH) filled with Ringer's solution. A first cut around 170 μm deep exposed the cell bodies from olfactory receptor neurons at the slice surface. After a second cut (approximately 200 µm deeper), the slice was transferred into a recording chamber filled with Ringer's solution.

### Selection of amino acid-sensitive cells in slices for patch-clamp recordings

To locate amino-acid-sensitive neurons, slices were stained with the fluorescent calcium dye Fluo-8-AM (50 µg, Molecular probes) dissolved in DMSO (Merck Millipore; final concentration < 0.5%) and Pluronic acid F-127 (Sigma-Aldrich; final concentration < 1%). To prevent destaining of the tissue via multidrug resistance receptors, the MDR-P-Inhibitor MK-571 (Enzo Life Sciences) was added^43,44^. Slices were incubated with the staining solution for at least 30 min. For the actual location of amino-acid-responsive cells, time series were acquired with either a laser scanning microscope (LSM 780, ZEISS) or a custom-built line illumination microscope, under the stimulation with a single amino acid (10 or 100 µM). After selecting an amino-acid-responsive ORN with fluorescence microscopy, wide field microscopy was used to find and patch the targeted cell body.

### Patch-clamp recordings from amino-acid-sensitive receptor neurons

Pipettes for patch-clamp experiments (Ø 1–2 μm, 5–7 MΩ) were pulled from borosilicate glass capillaries (Hilgenberg) using a two-stage pipette puller (PC-10, Narishige). They were filled with Ringer's solution and fixed to the electrode holder, which was mounted on a micromanipulator and connected to a patch-clamp amplifier (EPC8, HEKA). After forming a seal (GΩ range) and setting the holding potential to − 70 mV, ORN activity patterns were recorded using Linux-based data acquisition programs written in C++  (open source). Spontaneous activities (basal firing rates) refer to activity measured in the absence of any odor stimulation.

To investigate ORN sensitivity, two complementary patch clamp experiments were performed. For recordings from the first experiment, an amino-acid-concentration series (0, 0.01, 0.1, 1, 10, 25 and 100 µM, inter-stimulus interval > 1 min) was applied through a funnel positioned closely to the olfactory epithelium^45^. Ringer's solution was administered before the first and after each amino acid application. For recordings from the second experiment, ORNs were stimulated twice using an amino-acid concentration of 10 µM and 1 µM, respectively. Subsequently, ORNs were stimulated repetitively with the same concentration (0, 0.01, 0.1, 1 or 10 µM).

### Statistical analysis

The distribution of ORNs with distinct odorant response thresholds was analyzed as follows. The EC-50 value up on stimulation (8.8 µM) was used as a separator. Due to the concentrations used (0.01, 0.1, 1, 10 and 100 µM), this resulted into two groups: ORNs responding to amino-acid concentrations of < = 1 µM (low response threshold) and ORNs responding to concentrations of = > 10 µM (high response threshold). We used the Mann–Whitney-*U* test to calculate whether distribution within groups were statistically different.

### Staining of axon terminals from olfactory receptor neurons for rapid 4D calcium imaging

To visualize ORN axon terminals in the olfactory bulb, we electroporated nostrils of tadpoles as previously reported^38,46^. Before electroporation we anesthetized tadpoles with 0.02% MS-222 (ethyl-3-aminobenzoate-methanesulfonate; Sigma-Aldrich)^26^. Pieces of Calcium Green-1 dextran crystals (CaGreen; MW: 3000 Da, Sigma-Aldrich) were placed into both nostrils. For electroporation of the lateral cluster, one electrode was positioned in the nostril and the other electrode on the ipsilateral skin between nostril and eyes. After three subsequent pulses (20 ms, 20 V, 1 Hz) electrodes were slightly repositioned before three additional pulses were delivered. After electroporation, animals were housed in the dark at room temperature. Food and fresh water were provided regularly. Animals were sacrificed following a recovery period of at least 3 days.

### Whole-mount preparation for rapid 4D calcium imaging

Whole-mount preparation^26,38^ was achieved as follows. First, the above described sample tissue block was positioned on a preparation dish with the ventral side of the tissue block facing up. Pins inserted between the olfactory nerves were used to provide stability. A few drops of Ringer's solution were added to prevent dehydration. With a pair of scissors, the meninges covering the ventral part of the brain were removed. The sample was then transferred to a recording chamber filled with Ringer's solution and stabilized by a frame made of platinum wire and nylon threads.

### Rapid 4D calcium imaging recordings of amino-acid-responsive glomeruli

A 488 nm laser (Sapphire; Coherent) was used to excite CaGreen. The lateral glomerular cluster^45,47^ was located using the resulting fluorescence. Then a two- or three-dimensional time series (objective: 63x, NA 1.0, pixel size: 280 × 280 nm) of the lateral cluster was recorded (> 200*200(*5) μm, 30–60 s, > 2 Hz). Synchronously, a mixture of amino acids (10 or 100 μM) was administered to spot the location of functional glomeruli. For validating the anatomy of functional glomeruli a detailed z-stack (> 20 μm, distance between planes 1 μm) was recorded subsequently.

Odor concentrations used for dose–response profiles covered a range from several nanomolars up to 100 µM. When using any kind of stimulation, z-stacks of CaGreen-stained glomeruli were recorded using the following imaging conditions: 10 consecutive z-planes were acquired for ca. 4 min at a frequency of 1.9 stacks/s.

Spontaneous fluctuations were recorded using an imaging rate of minimally 3.8 z-stacks/s. Imaging rates faster than 7.2 z-stacks/s only allowed short (< 1 min) recordings due to photo bleaching. An application with Ringer's solution counted as a negative control, further applications with high-concentrated amino acids as a positive control. During experiments, there was a continuous flow of Ringer's solution (250 ml/h).

### Image analysis

All data were analyzed using custom-written Matlab scripts and GUIs (Mathworks).

### Removal of image line artifacts

Individual pixel intensity offset variations of the sCMOS detector used in the line illumination microscope were obtained by averaging intensities in all dimensions perpendicular to the detector line (y,z,t). The resulting pixel fluctuations were then subtracted from the acquired images (Fig. 9, Step 1).

### Drift, morph and photobleaching correction

For reliable 3D image drift detection, a band-pass filtered version of the original data was used. Data was smoothed for noise reduction using a Gaussian filter (σ = 1 pixels in x and y dimensions) and a low-pass version of the data (σ = 20) was subtracted to avoid cross-correlations from inhomogeneous image intensity distributions. Pairwise shift vectors between the starting reference z-stack and each consecutive z-stack of the time series were obtained via normalized image cross-correlation using Fast Fourier Transform and subsequent sub-pixel Gaussian fitting of the resulting peak position. Back-shifted data was calculated using cubic interpolation of the original data at the shifted pixel positions.

After drift correction, slow tissue distortions over the range of several minutes were detected using a set of drift corrections in multiple subareas positioned on a 2D triangular grid (grid point distance of 12 pixels for area sizes of 24 × 24 pixels, see Fig. 5C for exaggerated shift vectors) and sampled at broad temporal intervals (every 20 time points). Shift vectors were interpolated into a pixel-resolution shift vector field using weighted Gaussian interpolation (σ = 12 pixels), with the maximum cross-correlation coefficient of a grid point being used as a weight factor to only include image areas containing sufficient signal. The resulting morph vector field was then used for morphing correction using per-pixel cubic interpolation. Slow photobleaching was detected using per-pixel linear regression and subtracted from the data (corrections summarized in Fig. 9, step 2).

### Neighborhood correlation and binning

For the extraction of meaningful calcium traces, a neighborhood correlation map was calculated by cross-correlating the time trace of each pixel with the average of the 6 adjacent pixels in 3D. While pixels containing only background (static) signal or no signals are uncorrelated with their neighbors, pixels representing dynamic calcium changes are typically highly correlated due to the size of calcium compartments and the point spread function of the microscope being larger than one pixel. The neighborhood correlation map was then used as per-pixel-weights for binning the data into 3 × 3 (x and y) bins, favoring the pixels containing meaningful signal and improving overall signal/noise (Fig. 9, step 3).

### Calcium trace extraction

The resulting binned time traces were analyzed with the standard Matlab k-means clustering algorithm (typically k = 5) to reduce the number of different traces. Bin traces were assigned to their nearest clusters and subsequently averaged (Fig. 9, step 5 and 6). From the resulting traces, only the qualitatively different traces were manually selected.

### Activity correlation imaging

The selected traces were used as reference traces for pixel-wise temporal normalized correlation to the non-binned data, similar to^22^ (Fig. 9, step 7). Resulting correlation maps were combined by selecting, for each pixel, the highest-correlating map and assigning its chosen color. Pixel intensities were set to be proportional to the respective correlation coefficient. Maps of distinct components were manually merged (Fig. 9, step 8).

### Software used for composition of figures

Figure panels showing data have been composed with Matlab (2016b and 2017b, https://de.mathworks.com/products/get-matlab.html).

Figure panels were further composited with Adobe Illustrator CS6 (version 16.0.0, https://www.adobe.com/de/products/illustrator.html). Text annotations and additional annotations like arrows, lines, rectangles and boxes, were added if necessary.

## Supplementary Information

Supplementary Figures.

## Data Availability

The datasets generated during and/or analysed during the current study are available from the corresponding author on reasonable request.
