# Supplementary material for: Resolving different presynaptic activity patterns within single olfactory glomeruli of Xenopus laevis larvae
Source: Sci Rep. 2021 Jul 9;11:14258. doi: 10.1038/s41598-021-93677-9 (PMC8270923; doi:10.1038/s41598-021-93677-9)
Supplement: Supplementary file 1 — Supplementary Figures. [file 41598_2021_93677_MOESM1_ESM.docx]

**Resolving different presynaptic activity patterns within single olfactory glomeruli of *Xenopus laevis* larvae**

Rodi Topci **^1,2+^**, Mihai Alevra **^1,2,3+^ ,** Erik H.U. Rauf **^1,2^**, Daniëlle de Jong-Bolm * **^1,2,3^**

*1 Institute for Neurophysiology and cellular Biophysics, University of Göttingen Medical Center, 37073 Göttingen, Germany*

*2 Department of Neuro- and Sensory physiology, University of Göttingen Medical Center, 37073 Germany*

*3 Center Nanoscale Microscopy and Molecular Physiology of the Brain (CNMPB), Göttingen, Germany*

* [*danielle.dejong@med.uni-goettingen.de*](mailto:danielle.dejong@med.uni-goettingen.de)

**^+^** these authors contributed equally to this work


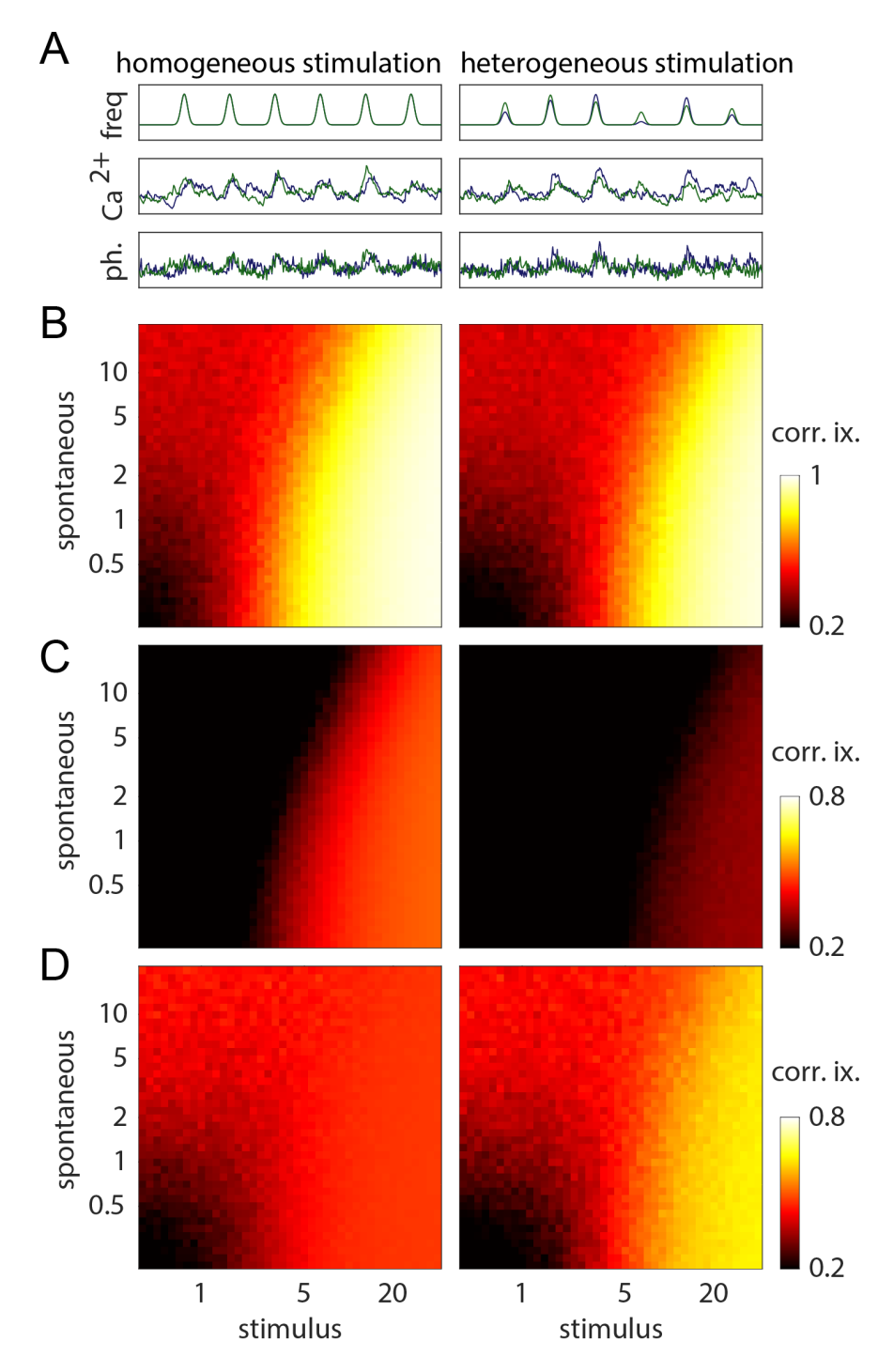
Supplementary Figure S1

**Supplementary Figure S1: Simulation of ROI correlation contrast for homogeneous and heterogeneous stimulation.** **A:** Top plots: olfactory stimulation amplitude over time, modeled as six gaussian-shaped peaks with either identical amplitude (“homogeneous stimulation”, left column) or varying amplitudes for each ROI (“heterogeneous stimulation”, right column, resembling different ROI sensitivities or other possible properties). Middle plot: simulated Ca^2+^ activity from pixels of two example ROIs. Lower plot: simulated detector signal from corresponding pixels. **B:** Intra-ROI correlation and its dependence on simulation parameters. Cross-correlation coefficients between traces of *the same* ROIs are averaged and displayed as color-coded pixels. The pixel location indicates simulation parameters used: on y axis the spontaneous firing rate (41 values logarithmically distributed between 0.2 Hz and 20 Hz), on x axis the peak stimulation firing rate (41 values logarithmically distributed between 0.4 and 40 Hz). Correlation generally increases with both spontaneous and stimulated firing rates. **C:** Inter-ROI correlations. Cross-correlation coefficients between traces from *different* ROIs are averaged and shown as in B. Correlations generally increase with stimulated, but only slightly with spontaneous activity (as it is less correlated between ROIs). **D:** Difference between B and C shows ROI correlation contrast. ROI contrast initially increases but saturates with spontaneous as well with stimulated activity in case of homogeneous stimulation, but further increases with stimulated activity if stimulated heterogeneously (yellow area on the right).

Supplementary Figure S2


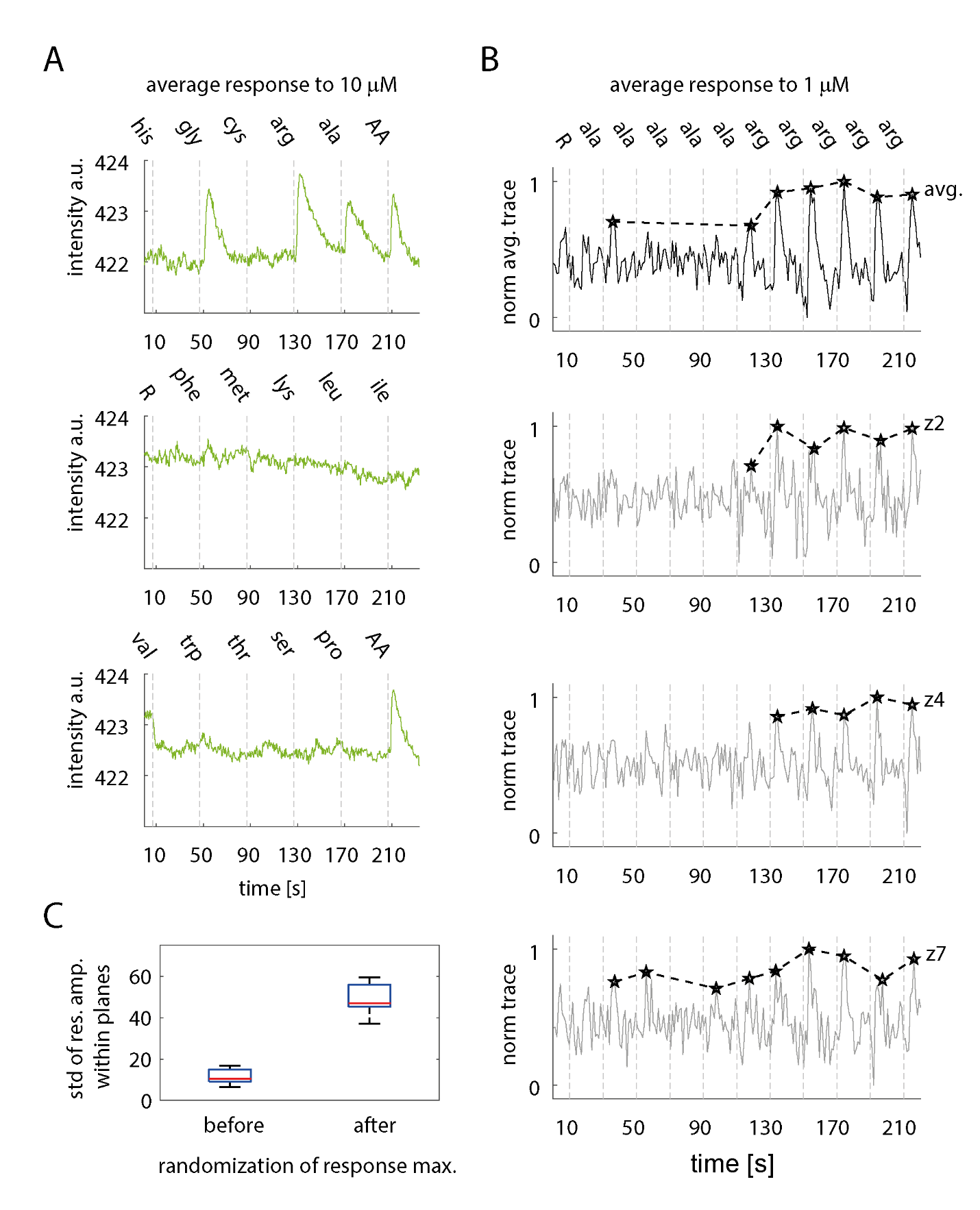


**Supplementary Figure S2: Glomerular responses to single amino acids. A:** raw traces from recordings under stimulation with single amino acids having a concentration of 10 µM. This glomerulus (for maximal projection and typical responses see Fig. 6) is responsive to multiple amino acids. L-alanine and L-arginine are chosen for subsequent stimulation using concentrations of 1 µM, traces are shown in D. **B:** Average response trace and response traces for single z-planes, both normalized to the maximum response. Responses maxima are indicated with a . **C:** A randomization of response maxima over z-planes, results in an increased response variation.

**
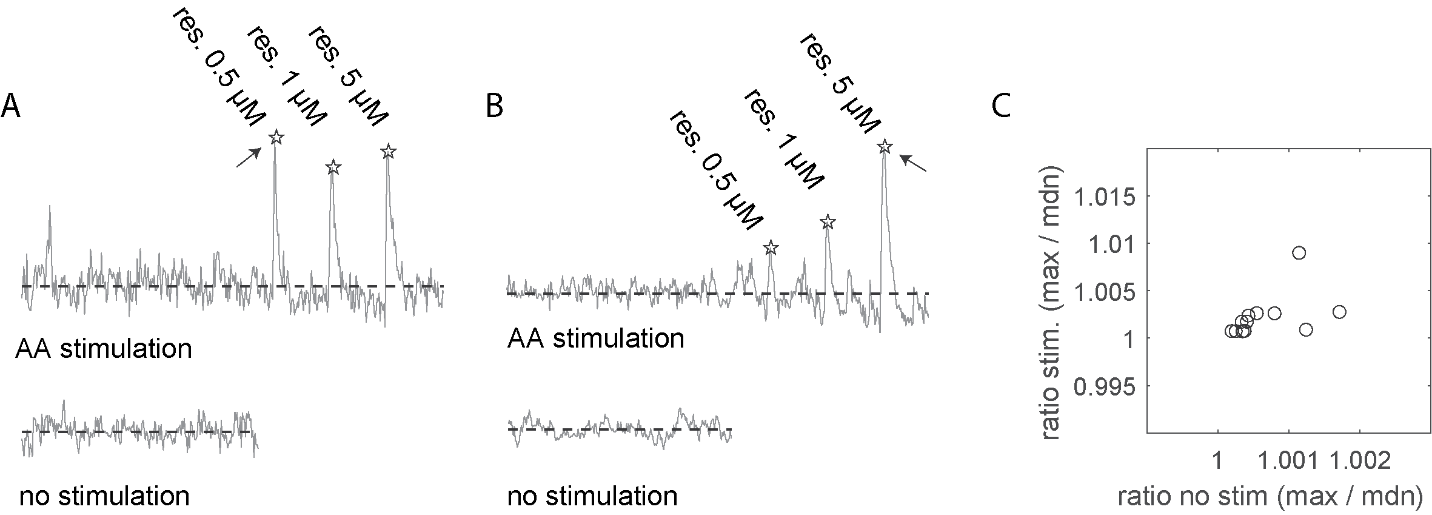
**Supplementary Figure S3

**Suppl. Figure S3: Ratios between maximum and median fluorescence intensities. A, B:** traces from exemplary glomeruli under AA stimulation or no stimulation. Stars are indicating local stimulus-induced response maxima. Of all stimulus-induced responses, arrows indicate the maximum response. Ratios between trace maxima and trace medians (mdn, dashed lines) are calculated. For “no stimulation” traces, maxima values used for ratios correspond to the mean of the 10 highest intensities. **C:** Comparison between ratios (max / mdn) from AA stimulation traces and no stimulation traces.


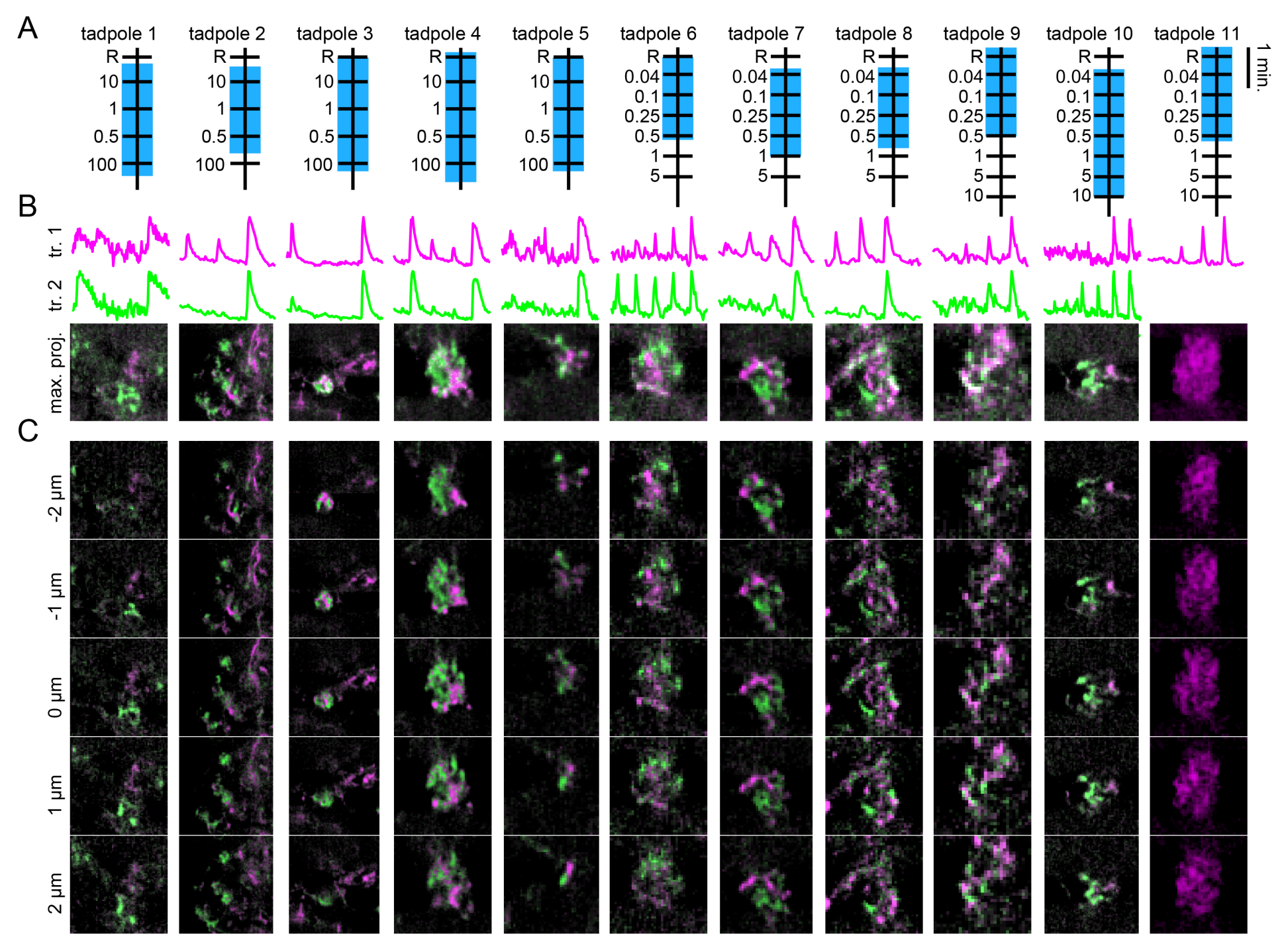
Supplementary Figure S4

**Supplementary Figure S4: Examples of two presynaptic components with distinct dynamic ranges.
A-C:** Each column represents one example. **A:** Experimental time line of recording. The vertical line in black indicates the length of the recording. The horizontal lines illustrate the stimulus type used and stimulus application time. The blue area marks the time window containing heterogeneous responses which are used for the auto detection of ROIs. **B:** Example traces in magenta and green, reflecting distinct dynamic ranges and their corresponding correlation maps. **C**: color projections of activity correlation maps. Each map was generated using the traces shown in B as reference traces.

Supplementary Figure S5


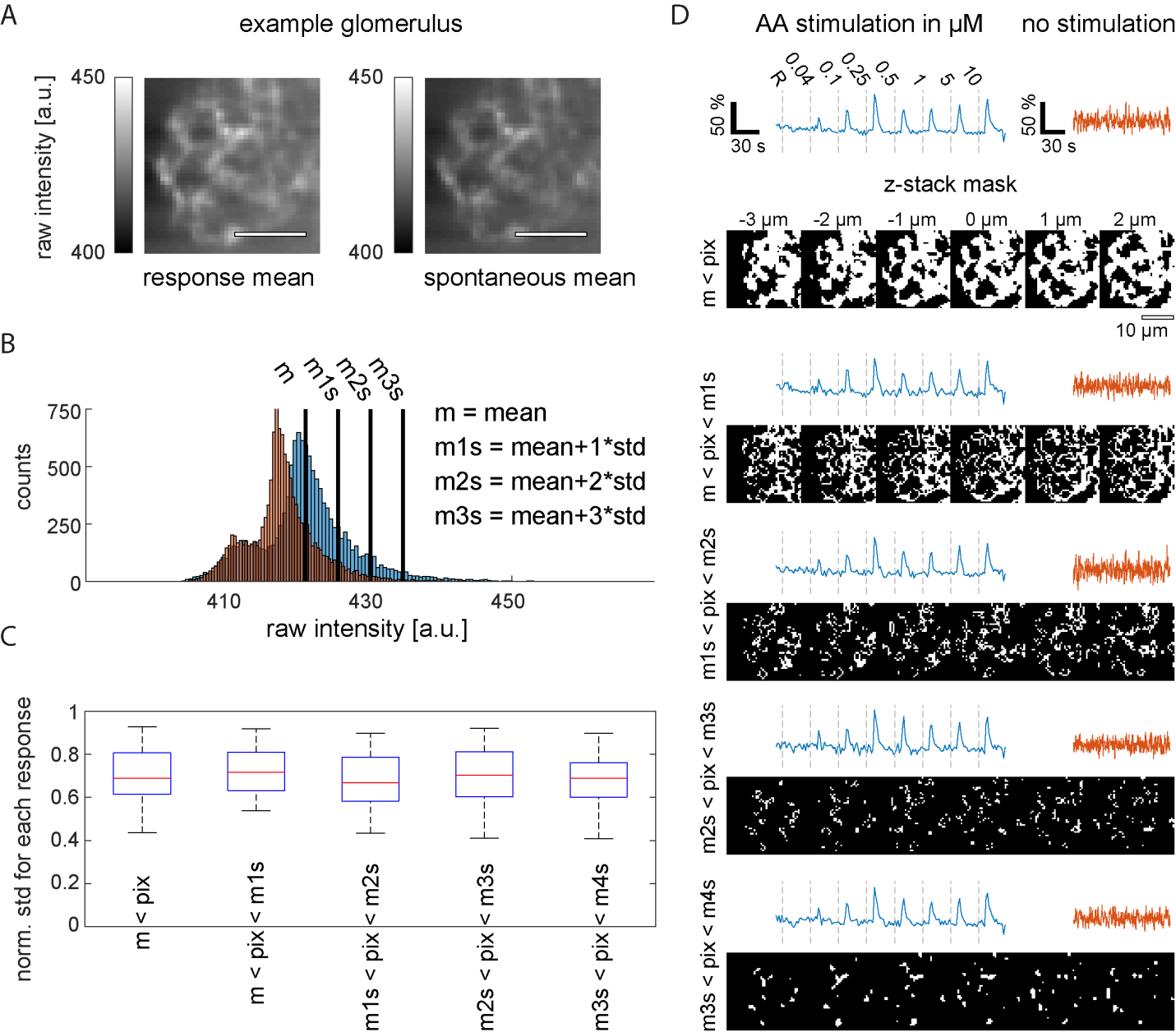


**Supplementary Figure S5: Regions categorized by fluorescence intensity levels have very similar responses.** **A:** Fluorescence intensities from two recordings of an example glomerulus, either under stimulation or not. **B:** Histogram of image intensities in A. Black vertical ms lines indicate the threshold values used for a comparison between regions with distinct fluorescence intensities. **C:** Boxplots representing normalized standard deviations of response maximum amplitudes (D). Mean standard deviations are indicated by the red lines. Number of pixels for each boxplot group from left to right: 5820 pixels, 3500 pixels, 1376 pixels, 562 pixels and 382 pixels. For this typical experiment, there was no difference between groups (Kruskall-Wallis test p = 0.95). **D:** Masks reveal pixels having different ranges of intensities. For recordings under stimulation (in blue) or no stimulation (in red) mean activity traces are shown. All scale bars shown represent 10 µm.
